# Population differentiation in allele frequencies of obesity-associated SNPs

**DOI:** 10.1186/s12864-017-4262-9

**Published:** 2017-11-10

**Authors:** Linyong Mao, Yayin Fang, Michael Campbell, William M. Southerland

**Affiliations:** 1 Department of Biochemistry and Molecular Biology, Howard University College of Medicine, 520 W Street NW, Washington, DC 20059 USA; 2 Department of Biology, Howard University, 415 College Street NW, Washington, 20059 DC USA

**Keywords:** Obesity, Gwas, Snp, Allele frequency, Population differentiation, Fto, Composite genetic risk score

## Abstract

**Background:**

Obesity is emerging as a global health problem, with more than one-third of the world’s adult population being overweight or obese. In this study, we investigated worldwide population differentiation in allele frequencies of obesity-associated SNPs (single nucleotide polymorphisms).

**Results:**

We collected a total of 225 obesity-associated SNPs from a public database. Their population-level allele frequencies were derived based on the genotype data from 1000 Genomes Project (phase 3). We used hypergeometric model to assess whether the effect allele at a given SNP is significantly enriched or depleted in each of the 26 populations surveyed in the 1000 Genomes Project with respect to the overall pooled population. Our results indicate that 195 out of 225 SNPs (86.7%) possess effect alleles significantly enriched or depleted in at least one of the 26 populations. Populations within the same continental group exhibit similar allele enrichment/depletion patterns whereas inter-continental populations show distinct patterns. Among the 225 SNPs, 15 SNPs cluster in the first intron region of the *FTO* gene, which is a major gene associated with body-mass index (BMI) and fat mass. African populations exhibit much smaller blocks of LD (linkage disequilibrium) among these15 SNPs while European and Asian populations have larger blocks. To estimate the cumulative effect of all variants associated with obesity, we developed the personal composite genetic risk score for obesity. Our results indicate that the East Asian populations have the lowest averages of the composite risk scores, whereas three European populations have the highest averages. In addition, the population-level average of composite genetic risk scores is significantly correlated (R^2^ = 0.35, *P* = 0.0060) with obesity prevalence.

**Conclusions:**

We have detected substantial population differentiation in allele frequencies of obesity-associated SNPs. The results will help elucidate the genetic basis which may contribute to population disparities in obesity prevalence.

**Electronic supplementary material:**

The online version of this article (10.1186/s12864-017-4262-9) contains supplementary material, which is available to authorized users.

## Background

Obesity is emerging as a global health problem, with more than one-third of the world’s adult population being overweight or obese [1]. Many serious health conditions are linked to obesity, including diabetes, hypertension, cardiovascular disease, and certain cancers [2–5]. It was estimated that overweight and obesity caused 3.4 million deaths in 2010 [6]. The serious public health burden of overweight and obesity makes it imperative to understand their underlying genetic and environmental causes.

**Fig. 1:**
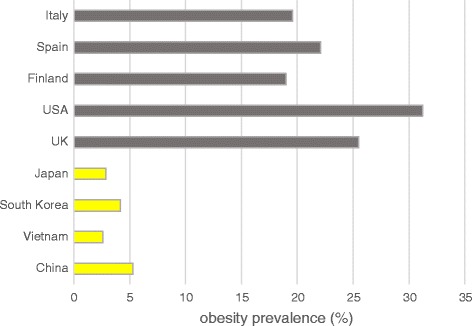
Obesity (BMI ≥ 30 kg/m^2^) prevalence by country. The data is for both sexes with ages greater than or equal to 18. The data was surveyed by WHO in 2010. Yellow for East Asian countries, black for European countries and USA

People of certain racial and ethnic groups are more (or less) likely to become obese. For example, based on the survey results from the World Health Organization (WHO), East Asian countries assumed much lower obesity rate than European countries and USA (Fig. 1). We hypothesized that the genetic factor may play a role in population disparities in the obesity prevalence. Recent genome-wide association studies (GWAS) have identified alleles in common variants that increased the risk of obesity [7]. However, these effect alleles may have different frequencies in different geographic regions due to genetic drift or natural selection [8–16], which may contribute to differences in the obesity prevalence between populations. Myles et al*.* studied 25 SNPs (single nucleotide polymorphisms) associated with 6 complex human diseases, and they proposed that SNPs with substantial variations in allele frequencies across populations might contribute to differences in disease prevalence among those populations [9]. Mattei et al. studied 101 SNPs in 30 genes involved in major metabolic and disease-relevant pathways in Puerto Ricans and compared them to similarly aged non-Hispanic whites (NHW) [8]. They found that, for the majority of SNPs having significantly different allele distributions between the two populations, Puerto Ricans carried risk alleles in higher frequency and protective alleles in lower frequency than NHW. Corona et al. found that differences in genetic dispositions to several diseases between different populations are beyond what is expected by genetic drift alone [10]. For example, the study demonstrated that populations from East Asia and the Americas have lower genetic risk for type 2 diabetes than those from Africa and Europe based on an analysis of 16 disease-associated SNPs.

Recent studies have also reported population differentiations in allele frequencies of obesity-associated SNPs. Adeyemo et al. investigated 29 obesity-associated SNPs for their allele frequency variations among 11 populations by using genotype data from the International HapMap Project [17]. Harnessing genome-wide SNP results of 938 individuals from the Human Genome Diversity Panel, Klimentidis et al. examined the worldwide population differentiation pattern in the genomic regions surrounding 16 obesity risk alleles [14]. Although Wang et al. tested signals of positive selection at 115 BMI (body mass index) – associated SNPs among 14 populations of 1000 Genomes Project (phase 1 data), they did not specifically study relationships between allele frequencies and obesity prevalence [18]. In this study, we compiled a comprehensive set of 225 obesity-associated SNPs and assessed their population differentiations in allele frequencies by utilizing 1000 Genomes Project phase 3 data [19], which identifies genetic variants among 26 worldwide populations. We also constructed the composite genetic risk score for obesity at both the individual and population levels, and tested the correlation between the population-level average of composite risk scores and obesity prevalence.

## Methods

One thousand Genomes Project surveys genetic variations among 2504 individuals from 26 worldwide populations [19]. These 26 populations can be grouped into Africa (AFR), East Asia (EAS), Europe (EUR), South Asia (SAS), and the Americas (AMR) based on their geographical locations and ancestries (Table 1). The number of individuals surveyed in each of the 26 populations ranges from 61 to 113 with an average of 96, while the number of individuals per continental group ranges from 347 to 661 with an average of 501. The phase 3 genotype data of these 2504 individuals was downloaded from ftp://ftp.1000genomes.ebi.ac.uk/vol1/ftp/release/20130502/. The variant coordinates were based on the human genome assembly GRCh37. All alleles in the 1000 Genomes Project were reported on the forward strand.

**Table 1:** 26 populations surveyed in the 1000 Genomes Project

population	population abbreviation	Continental group	n
African Caribbean in Barbados	ACB	African (AFR)	96
African Ancestry in Southwest US	ASW	African (AFR)	61
Bengali in Bangladesh	BEB	South Asian (SAS)	86
Chinese Dai in Xishuangbanna, China	CDX	East Asian (EAS)	93
Utah residents with Northern and Western European ancestry	CEU	European (EUR)	99
Han Chinese in Beijing, China	CHB	East Asian (EAS)	103
Southern Han Chinese, China	CHS	East Asian (EAS)	105
Colombian in Medellin, Colombia	CLM	American (AMR)	94
Esan in Nigeria	ESN	African (AFR)	99
Finnish in Finland	FIN	European (EUR)	99
British in England and Scotland	GBR	European (EUR)	91
Gujarati Indian in Houston,TX	GIH	South Asian (SAS)	103
Gambian in Western Division, The Gambia	GWD	African (AFR)	113
Iberian populations in Spain	IBS	European (EUR)	107
Indian Telugu in the UK	ITU	South Asian (SAS)	102
Japanese in Tokyo, Japan	JPT	East Asian (EAS)	104
Kinh in Ho Chi Minh City, Vietnam	KHV	East Asian (EAS)	99
Luhya in Webuye, Kenya	LWK	African (AFR)	99
Mende in Sierra Leone	MSL	African (AFR)	85
Mexican Ancestry in Los Angeles, California	MXL	American (AMR)	64
Peruvian in Lima, Peru	PEL	American (AMR)	85
Punjabi in Lahore, Pakistan	PJL	South Asian (SAS)	96
Puerto Rican in Puerto Rico	PUR	American (AMR)	104
Sri Lankan Tamil in the UK	STU	South Asian (SAS)	102
Toscani in Italy	TSI	European (EUR)	107
Yoruba in Ibadan, Nigeria	YRI	African (AFR)	108

We searched the NHGRI-EBI GWAS Catalog (https://www.ebi.ac.uk/gwas/home, December 2015) for SNPs that were associated with at least one of the obesity related traits (*p*-value <9 × 10^−6^). The traits include BMI, obesity, obesity (early onset extreme), waist circumference and waist-hip ratio according to [7]. By examining the sign of beta-coefficient, whether the odds ratio for the effect allele is greater than one, and text description in the primary GWAS reports, we determined obesity risk (obesity-increasing) alleles for the obesity-associated SNPs. In addition, we checked whether an obesity effect (risk) allele stored in the GWAS Catalog is on the forward or reverse strand based on the content of primary GWAS reports. To retrieve population-level allele frequencies from the genotype data of 1000 Genomes Project, we converted nucleotide (i.e. effect allele) of an SNP to its complement if it was reported on the reverse strand in the GWAS Catalog.

We used hypergeometric test to assess if the effect (risk) allele of an obesity-associated SNP is significantly enriched or depleted (two separate tests) in each of the 26 populations with respect to the global population, which pulls all 26 populations together. Thus, for each SNP, 52 hypergeometric tests (2 × 26) were performed. With a total of 225 obesity SNPs retrieved from the GWAS Catalogue, we performed 11,700 statistical tests. To control a family-wise error rate (FWER) of 0.01, we used a raw *p*-value of 0.01/11700 = 8.55 × 10^−7^ as cutoff. In generating heatmaps to visualize allele enrichment/depletion patterns in different populations, the hypergeometric testing *p*-values were first log_10_ transformed. If the effect allele of an SNP is enriched in a population, then the negative of log_10_ of the enrichment p-value (a positive number) was used to represent the SNP in association with that population in a heatmap. On the other hand, if the allele of an SNP is depleted in a population, the value of log_10_ of the depletion p-value (a negative number) was used to represent the SNP for that population in the heatmap. We used dChip software [20] to perform hierarchical clustering based on enrichment/depletion *p*-values (log_10_ based) of effect alleles in populations. Centroid option was selected as the linkage method for clustering. The distance between two nodes is 1 – correlation. Thus, the minimal distance is zero when two nodes are perfectly correlated, and maximal distance is two when two nodes are negatively correlated.

Linkage disequilibrium statistics (r^2^) between a pair of SNPs was calculated using LDlink [21], which uses haplotype data from the 1000 Genomes Project.

We applied the following equation to calculate the composite genetic risk score for obesity,

1 $$ risk\_ score=\frac{\sum_{i=1}^I{X}_i\ }{2I} $$

where *I* refers to the number of obesity risk SNPs, and *X*_*i*_ refers to copies of risk alleles (*X*_*i*_ ∈ {0,1,2}) at the *i* ^*th*^ SNP. In one extreme case, if a person has two copies of risk alleles at each obesity SNP, then the person’s risk score will become 1. On the other hand, if a person has zero copy of risk alleles at each obesity SNP, then the person’s risk score will become 0. A person with the composite score of 1 has maximal possible genetic risk for obesity while a person with the score of 0 has the lowest possible genetic risk. If copies of effect alleles (0/1/2) are randomly assigned to each SNP, the expected value of the risk score will be 0.5. Although we collected 225 obesity SNPs, we only chose SNPs which have reached genome-wide significance (*P* < 5 × 10^−8^) in GWA studies to calculate the composite score, which resulted in 155 obesity risk SNPs. We applied the formula to calculate the composite genetic risk score for each individual present in the 1000 Genomes Project and then summarized the risk score at a population level (e.g. average, median).

Our formula does not carry weights for alleles, and it is possible that not all 155 obesity-associated SNPs used to calculate the composite genetic risk score are independent to each other. To address this concern, we performed the following analysis. From the 155 obesity-associated SNPs, we specifically targeted 32 of these SNPs to examine the per allele change in BMI (kg/m^2^) which were derived from an analysis of 249,796 individuals of European ancestry [22]. These 32 SNPs have known effect sizes and are considered to be independent since the pair-wise linkage disequilibrium (LD, *r* ^2^) was less than 0.1 and since they were separated by at least 1 Mb [22]. Let *S* be one of the 32 independent SNPs. We counted how many nearby SNPs, among the 155 SNPs, that are within 1 Mb from *S* (including *S* itself). We then computed the Pearson correlation coefficient between the effect size (kg/m^2^) of *S* and number of nearby SNPs.

The country-wise obesity (BMI ≥ 30 kg/m^2^) prevalence data was surveyed by WHO (World Health Organization) in 2010 (http://apps.who.int/gho/data/node.main.A900A?lang=en). We used the average of composite genetic risk scores for the population(s) residing in (or emigrating from) a country (Table 1) to correlate with the country’s obesity rate. Specifically, for countries with multiple populations profiled in 1000 Genomes Project, we pooled ESN (Esan in Nigeria) and YRI (Yoruba in Ibadan, Nigeria) populations to obtain the average of composite scores for Nigeria; we pooled CHB (Han Chinese in Beijing) and CHS (Southern Han Chinese) for China; and we pooled GIH (Gujarati Indian in Houston,TX) and ITU (Indian Telugu in the UK) for India. In addition, we used CEU (Utah residents with Northern and Western European ancestry) average of composite scores as an approximation for the USA. The WHO data did not include Puerto Rican obesity rate.

## Results

### Obesity alleles

We collected a total of 225 obesity-associated SNPs from the NHGRI-EBI GWAS Catalog [23] (Additional file 1: Table S1). The 225 obesity-associated SNPs originated from 29 GWA studies (Additional file 2: Table S2). Among them, 19 were performed in European populations, 3 in East Asians, 2 in South Asians, and 3 in Africans. The two remaining GWA studies were performed in mixed ethnic populations [24, 25]. Clearly, populations except Europeans were understudied.

**Fig. 2:**
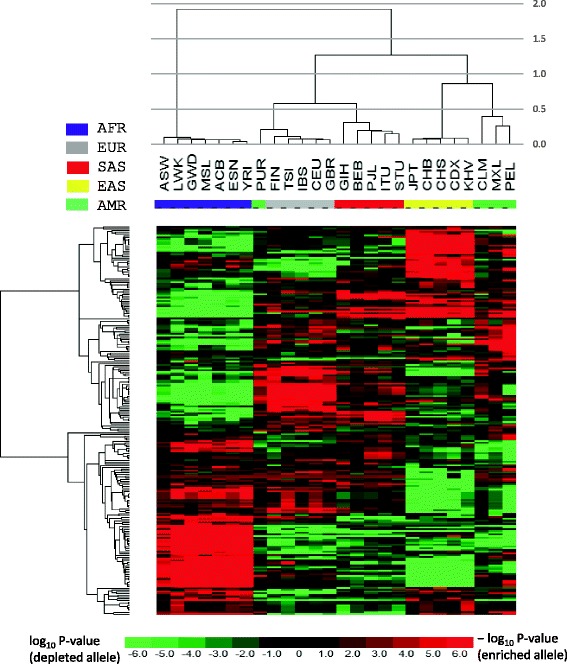
Heatmap showing how significantly the effect alleles are enriched or depleted in each population for the 225 obesity risk SNPs. Each row represents an SNP, and each column represents a population. The color bar above the heatmap shows continental group each population belongs to. Keys for populations can be found in Table 1. A cell in the heatmap is color coded according to the log10 of *P*-value, which tests the enrichment/depletion of an effect allele in a population in comparing with the overall average. If an effect allele is enriched, the cell is colored red based on the negative value of log_10_P, whereas if an effect allele is depleted, the cell is colored green based on the value of log_10_P

Following collecting the obesity-associated SNPs, we obtained their effect allele frequencies in each of the 26 populations (Additional file 1: Table S1) based on genotype information from the 1000 Genomes Project. We then tested, for each SNP, if the effect allele is enriched or depleted in each of the 26 populations in comparison with the overall population average. A heatmap (Fig. 2) shows how significantly the effect alleles were enriched or depleted across the 26 populations among 225 obesity risk SNPs. At the FWER-adjusted *p*-value of 0.01, among the 225 SNPs, the effect alleles of 145 SNPs were both significantly enriched in at least one population and significantly depleted in at least another population, 18 SNPs were significantly enriched in at least one population but not significantly depleted in any other population, and 32 SNPs were only significantly depleted in some population(s). Thus, 195 out of 225 SNPs (86.7%) were significantly enriched or depleted in at least one of the 26 populations. A hierarchical clustering of the 26 populations clusters the populations into their corresponding continental groups (Fig. 2) except the Puerto Rican population (PUR). The Puerto Rican population is sister to the European continental group but not clustered with the American group. However, this observation is consistent with the finding that the ancestral composition of the Puerto Rican population includes 57.2% European [26]. Studies also show that the Americas (AMR) is an admixture among European, East Asian and African ancestries [19]. We also observed that intra-continental populations were in general tightly clustered together whereas inter-continental populations show distinct allele enrichment/depletion patterns. The African continental group especially shows a negative correlation with the remaining populations in the hierarchical clustering tree. One possible explanation for these results is that continent-specific environmental factors may shape the allele abundance of obesity-associated SNPs in the ancestries of continental populations.

**Fig. 3:**
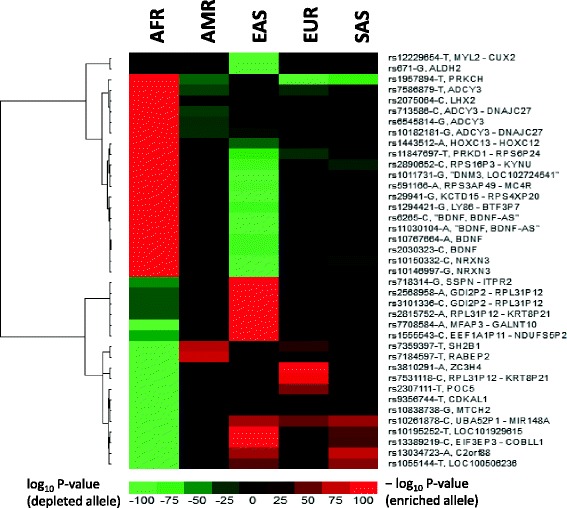
Heatmap showing how significantly the effect alleles are enriched or depleted in each continental group for the 39 obesity risk SNPs. SNP ID is shown for each row in the heatmap and followed by its obesity-increasing allele and neighboring (or containing) gene. See Table 2 for details

Because populations belonging to the same continental group exhibit similar patterns in allele enrichment/depletions, we decided to merge them to examine patterns at the continental level with benefits of larger sample sizes and simplification of pattern recognition. A heatmap (Fig. 3) visualizes how significantly the effect alleles were enriched or depleted in each continental group in comparison with the global average for a selected set of 39 obesity risk SNPs, which have enrichment or depletion *p*-values of at least 10^−100^ and have reached genome-wide significance (5 × 10^−8^) in GWA studies. The heatmap clearly shows that African and East Asian populations have the largest number of SNPs that exhibit the most significant allele frequency changes. In many cases, African and East Asian populations exhibit opposite directions in allele frequency changes – effect alleles were enriched in one population but depleted in the other. For example, SNP rs2030323, located in the intron of *BDNF* (brain derived neurotrophic factor) which encodes a member of the nerve growth factor family of proteins, has C/A alleles in which the C allele was tested in European [27] and East Asian [28] populations to increases obesity risks. The C allele has 77%, 51% and 95% frequencies in European, East Asian and African populations, respectively (Table 2). The allele frequency in Africans is almost twice of East Asians. In the Esan population in Nigeria (ESN), the C allele frequency has reached 99.5% (*n* = 99, Additional file 1: Table S1). In another contrasting example, rs7708584, approximately 27 kb upstream of *GALNT10* (polypeptide N-acetylgalactosaminyltransferase 10) whose protein product functions in the synthesis of mucin-type oligosaccharides [29], has A/G alleles in which the A allele was shown to increase BMI in African populations [30]. The A allele has 96% and 26% frequencies in East Asian and African populations, respectively. The allele frequency in East Asians is more than three times of Africans. In particular, in the Japanese population (JPT), the A allele frequency has reached 99.0% (*n* = 104). rs671, an SNP located in the coding region of *ALDH2* (aldehyde dehydrogenase 2) which encodes an enzyme of the major oxidative pathway of alcohol metabolism, is associated with BMI in East Asian population [31]. The BMI-increasing allele, G, has a frequency of 83% in East Asians while it is fixed (100%) in Europeans (*n* = 503). Thus, this SNP cannot be identified as an obesity-associated locus in the European population. rs29941 and rs7359397, two SNPs close to *KCTD15* (potassium channel tetramerization domain containing 15) and *SH2B1* (SH2B adaptor protein 1), respectively, were among the 39 SNPs (Table 2). KCTD15 inhibits neural crest formation during embryonic development [32], and *SH2B1* encodes the Src homology 2B adaptor protein 1, a protein mediates activation of various kinases and may function in cytokine and growth factor receptor signaling [33, 34]. These two genes were also found to be related to obesity with risk allele frequencies differing substantially between populations [14]. For rs7359397, its risk allele frequency in American group (47%) is 34 times of African group (1.4%). These results demonstrate extreme cases of population differentiation in obesity risk allele frequencies.

**Table 2:** Effect allele frequencies (EAF) of 39 obesity risk SNPs in continental groups

SNP ID	chr	Position^a^	function^b^	Nearby / containing gene^b^	Effect allele^c^	Other allele^c^	African EAF	American EAF	East Asian EAF	European EAF	South Asian EAF	Global EAF	GWAS *p*-value
rs1011731	1	172,346,548	intron	DNM3	G	A	0.90	0.36	0.13	0.42	0.43	0.48	1.0E-17
rs10146997	14	79,945,162	intron	NRXN3	G	A	0.41	0.20	0.00	0.21	0.11	0.20	5.0E-08
rs10150332	14	79,936,964	intron	NRXN3	C	T	0.44	0.20	0.00	0.21	0.11	0.21	3.0E-11
rs10182181	2	25,150,296	intergene	ADCY3 - DNAJC27	G	A	0.91	0.38	0.44	0.47	0.48	0.57	1.0E-17
rs10195252	2	165,513,091	intron	LOC101929615	T	C	0.20	0.74	0.90	0.56	0.79	0.60	2.0E-24
rs10261878	7	25,950,545	intergene	UBA52P1 - MIR148A	C	A	0.34	0.89	0.96	0.94	0.96	0.78	1.0E-10
rs1055144	7	25,871,109	ncRNA	LOC100506236	T	C	0.04	0.16	0.43	0.21	0.46	0.25	1.0E-24
rs10767664	11	27,725,986	intron	BDNF	A	T	0.95	0.83	0.51	0.77	0.70	0.76	5.0E-26
rs10838738	11	47,663,049	intron	MTCH2	G	A	0.05	0.40	0.28	0.35	0.33	0.26	5.0E-09
rs11030104	11	27,684,517	intron;intron	BDNF, BDNF-AS	A	G	0.98	0.83	0.51	0.78	0.74	0.78	2.0E-20
rs11847697	14	30,515,112	intergene	PRKD1 - RPS6P24	T	C	0.41	0.05	0.00	0.05	0.11	0.15	6.0E-11
rs12229654	12	111,414,461	intergene	MYL2 - CUX2	T	G	1.00	1.00	0.84	1.00	1.00	0.97	5.0E-09
rs1294421	6	6,743,149	intergene	LY86 - BTF3P7	G	T	0.77	0.50	0.26	0.61	0.40	0.53	2.0E-17
rs13034723	2	190,985,680	intron	C2orf88	A	G	0.06	0.45	0.69	0.46	0.70	0.45	2.0E-08
rs13389219	2	165,528,876	intergene	EIF3EP3 - COBLL1	C	T	0.20	0.74	0.90	0.56	0.78	0.60	3.0E-08
rs1443512	12	54,342,684	intergene	HOXC13 - HOXC12	A	C	0.64	0.21	0.17	0.22	0.32	0.34	6.0E-17
rs1555543	1	96,944,797	intergene	EEF1A1P11 - NDUFS5P2	C	A	0.39	0.57	0.88	0.58	0.51	0.58	4.0E-10
rs1957894	14	61,908,111	intron	PRKCH	T	G	0.77	0.13	0.41	0.08	0.11	0.34	3.0E-10
rs2030323	11	27,728,539	intron	BDNF	C	A	0.95	0.83	0.51	0.77	0.70	0.76	3.0E-22
rs2075064	9	126,783,847	intron	LHX2	C	T	0.93	0.54	0.58	0.56	0.65	0.68	2.0E-08
rs2307111	5	75,003,678	missense	POC5	T	C	0.08	0.52	0.47	0.60	0.40	0.38	3.0E-12
rs2568958	1	72,765,116	intergene	GDI2P2 - RPL31P12	A	G	0.53	0.70	0.93	0.64	0.62	0.68	4.0E-16
rs2815752	1	72,812,440	intergene	RPL31P12 - KRT8P21	A	G	0.53	0.70	0.93	0.64	0.62	0.68	2.0E-22
rs2890652	2	142,959,931	intergene	RPS16P3 - KYNU	C	T	0.38	0.09	0.00	0.17	0.06	0.16	1.0E-10
rs29941	19	34,309,532	intergene	KCTD15 - RPS4XP20	G	A	0.85	0.61	0.24	0.67	0.62	0.61	7.0E-12
rs3101336	1	72,751,185	intergene	GDI2P2 - RPL31P12	C	T	0.53	0.70	0.93	0.64	0.62	0.68	1.0E-13
rs3810291	19	47,569,003	3′-UTR	ZC3H4	A	G	0.09	0.51	0.27	0.66	0.40	0.36	2.0E-12
rs591166	18	57,841,589	intergene	RPS3AP49 - MC4R	A	T	0.82	0.38	0.22	0.43	0.49	0.50	7.0E-14
rs6265	11	27,679,916	missense;ncRNA	BDNF, BDNF-AS	C	T	0.99	0.85	0.51	0.80	0.80	0.80	5.0E-10
rs6545814	2	25,131,316	intron	ADCY3	G	A	0.85	0.35	0.42	0.43	0.46	0.54	1.0E-13
rs671	12	112,241,766	missense	ALDH2	G	A	1.00	1.00	0.83	1.00	1.00	0.96	3.0E-11
rs713586	2	25,158,008	intergene	ADCY3 - DNAJC27	C	T	0.92	0.38	0.48	0.47	0.48	0.58	6.0E-22
rs718314	12	26,453,283	intergene	SSPN - ITPR2	G	A	0.19	0.49	0.70	0.24	0.24	0.35	1.0E-17
rs7184597	16	28,921,809	intron	RABEP2	T	C	0.01	0.45	0.12	0.28	0.16	0.18	7.0E-09
rs7359397	16	28,885,659	downstream of SH2B1	SH2B1	T	C	0.01	0.47	0.12	0.33	0.19	0.19	2.0E-20
rs7531118	1	72,837,239	intergene	RPL31P12 - KRT8P21	C	T	0.05	0.38	0.26	0.55	0.29	0.29	2.0E-17
rs7586879	2	25,116,977	intron	ADCY3	T	C	0.86	0.29	0.37	0.35	0.43	0.50	4.0E-08
rs7708584	5	153,543,466	intergene	MFAP3 - GALNT10	A	G	0.26	0.63	0.96	0.44	0.47	0.53	5.0E-14
rs9356744	6	20,685,486	intron	CDKAL1	T	C	0.30	0.67	0.61	0.68	0.71	0.57	5.0E-13

^a^The variant coordinates were based on the human genome assembly GRCh37

^b^Annotations were obtained from the NHGRI-EBI GWAS Catalog

^c^All alleles were reported on the forward strand

### *FTO* SNPs

**Fig. 4:**
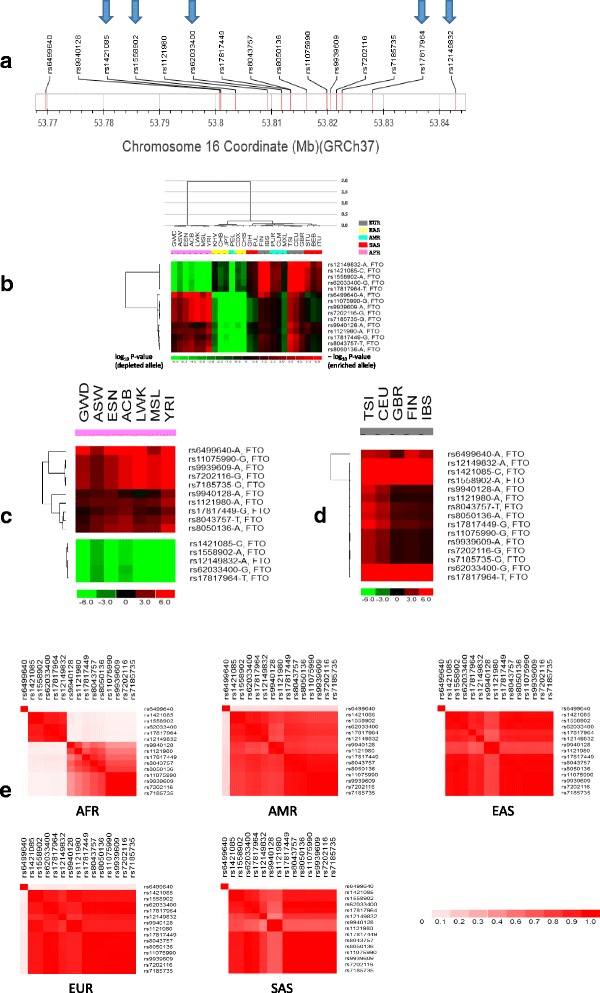
*FTO* SNPs. (**a**) Physical positions of 15 *FTO* SNPs on chromosome 16. (**b**) Heatmap showing how significantly the effect alleles of *FTO* SNPs are enriched or depleted in each of the 26 populations in comparison with the overall average. SNP ID is shown for each row of the heatmap and followed by its obesity-increasing allele. The 15 *FTO* SNPs were divided into two clades. The first clade has 5 members, which are indicated by arrows in **a**. (**c**) Heatmap for populations of African ancestry. 5 SNPs with depleted alleles (green color) formed the first major LD block, whereas the 9 SNPs with enriched alleles (red) except rs6499640 formed the second major LD block in African group (**e**). Also refer to the main text. (**d**) Heatmap for populations of European ancestry. (**e**) Five heatmap matrixes of pairwise linkage disequilibrium statistics (r^2^) for five continental groups, respectively. Each cell in the heatmap represents correlation (r^2^) between a pair of SNPs

Although the investigation of the molecular function of *FTO* (fat mass and obesity associated) has not led to conclusive results [35, 36], the gene plays a role in controlling feeding behavior and energy expenditure [37]. An analysis of 249,796 individuals of European ancestry identified 32 SNPs that were significantly associated with BMI (*P* < 5 × 10^−8^) [22]. Among them, the *FTO* SNP, rs1558902, accounted for the largest proportion of the variance. European adults who carried two copies of the risk allele in the *FTO* SNP, rs9939609, weighed about 3 kg more and had 1.67-fold increased odds of obesity in comparison with those with no copies of this allele [38]. Among the 225 obesity-associated SNPs collected in this study, 15 are positioned in the *FTO* locus (Fig. 4a), and all of them are within the first intron of *FTO* and increase obesity risk. In the hierarchical clustering tree of all obesity risk SNPs (Fig. 2), these 15 SNPs were exclusively grouped into two monophyletic clades (one clade consisting of 5 SNPs and the other clade consisting of 10 SNPs). These two clades exhibit distinct allele enrichment/depletion patterns mainly due to the African populations (Fig. 4b). In the 5-member clade, the effect alleles of 5 *FTO* SNPs were depleted in the African populations in comparison with the overall population average; whereas in the 10-member clade, the effect alleles of 10 *FTO* SNPs were enriched in the African populations. Surprisingly, the set of 5 *FTO* SNPs depleted in the African populations is not physically separated from the other 10 SNPs on the chromosome (Fig. 4a), instead, they are intertwined. In contrast to the African populations, the 15 *FTO* risk alleles were unanimously enriched in the European populations, but they were depleted in the East Asian populations as well as the Peru population (PEL).

We further analyzed LD (linkage disequilibrium) patterns between the *FTO* SNPs in five continental groups, respectively (Fig. 4e). rs6499640 shows no or weak LD signal with the other 14 SNPs in all five continental groups, which is in accordance with its chromosomal position separated from the other 14 SNPs (Fig. 4a). In the hierarchical clustering tree of *FTO* SNPs across 26 populations (Fig. 4b), rs6499640 formed a branch by itself with distinct allele enrichment/depletion pattern. The hierarchical clustering of SNPs within African (Fig. 4c) and European (Fig. 4d) continental group further demonstrates the unique allele pattern of rs6499640. Except rs6499640, the other 14 SNPs fall in a region of very strong LD in the European continental group and moderately strong LD in American, East Asian, and South Asian groups (Fig. 4e). However, these 14 SNPs were apparently split into two major LD blocks in the African group, as they were divided into two clades in the SNP hierarchical tree (Fig. 4b) with opposite allele enrichment/depletion patterns in the African populations. The five SNPs in the first major LD block of the African group are strongly linked to each other, whereas the second major LD block containing nine SNPs is more fragmented. These nine SNPs were also partitioned into multiple branches in the hierarchical clustering tree of the SNPs within African populations (Fig. 4c). For example, the four SNPs (rs11075990, rs9939609, rs7202116 and rs7185735) comprising a branch with a small branch height corresponded to a relatively strong sub-block within the second major LD block of African group. In contrast, all 14 SNPs formed a low-height branch in the hierarchical clustering tree of the European populations (Fig. 4d) and fell in a region of strong LD. Thus, the African populations have much smaller blocks of LD than the other populations in the *FTO* locus, indicating that the African populations are more genetically diverse in this genomic region.

Although those 15 obesity-associated SNPs are located within the first intron of *FTO*, it should be noted that some of the variants can form long-range functional connections with the homeobox gene *IRX3* [39], which is a half-megabase downstream of the variants.

### Composite genetic risk score

**Fig. 5:**
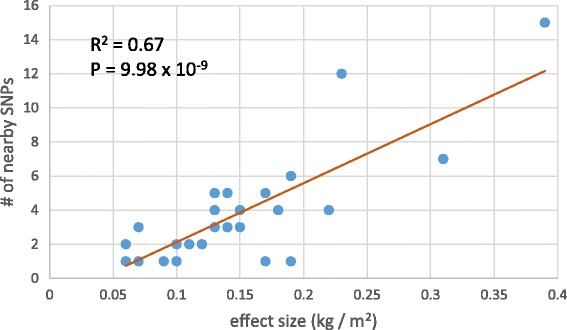
Correlation between effect sizes of independent SNPs and number of neighboring SNPs. The neighboring SNP refers to an SNP which is within 1 Mb from an independent SNP and which is included to compute the composite genetic risk score

We developed a mathematical formula (equation 1) to calculate the composite genetic risk score based on copies of effect alleles at obesity-associated SNPs. Although the majority of obesity-associated SNPs were detected from genome-wide association studies of European populations (Additional file 2: Table S2), we assumed that these variants would also be associated with the condition in non-European populations. This assumption is somewhat validated by a study which found that allelic associations from a significant majority of GWAS-identified variants can be replicated in non-European populations and the associations are in the same direction as in European populations [40]. In the equation, we also assumed that each variant contributed equally to the genetic risk score. Different variants should carry different weights in a more rational representation of the genetic risk. However, not all variants have known effect sizes, and these effect sizes were mainly estimated from European populations. It would not be appropriate to extrapolate the European-derived effect sizes to other populations because of their inconsistency across different populations [40]. Nevertheless, we found a significantly positive correlation (R^2^ = 0.67, *P* = 9.98 × 10^−9^, Fig. 5) between the number of SNPs clustered within a narrow genomic region and effect size of an independent SNP representing that genomic region (see Methods). Thus, the inclusion of clustered SNPs, some of which may be co-inherited in certain populations, in our calculation of the composite score could compensate SNP-specific effect size to some degree.

**Fig. 6:**
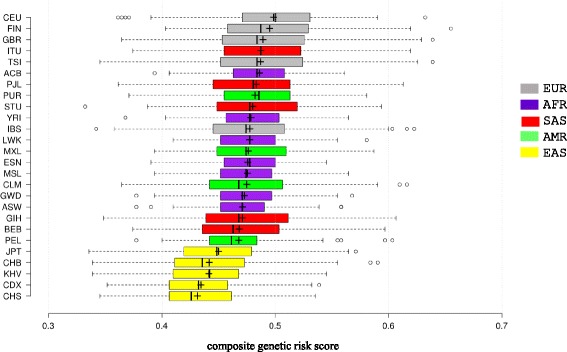
Distribution of composite genetic risk scores for obesity. For each population, plus symbol indicates average, center line in the box plot shows the median, box boundaries indicate the 25th and 75th percentiles, whiskers extend 1.5 times the interquartile range from the 25th and 75th percentiles, and outliers are represented by circles. The order of populations depicted in the figure is sorted according to their averages of composite scores. Plotted using BoxPlotR [48]

We used all 155 SNPs, which have reached genome-wide significance (*P* < 5 × 10^−8^) in GWA studies (Methods), to calculate the composite genetic risk score for each person present in the 1000 Genomes Project (*N* = 2504). Their composite scores range from 0.33 to 0.65 with an average of 0.47 (STD = 0.049), which is close to the expected value (0.5) if copies of effect alleles are randomly assigned to each SNP. Distributions of composite scores for the 26 populations are shown in Fig. 6. Clearly, the five East Asian populations have the lowest average and median of the composite scores among the five continental groups.

**Fig. 7:**
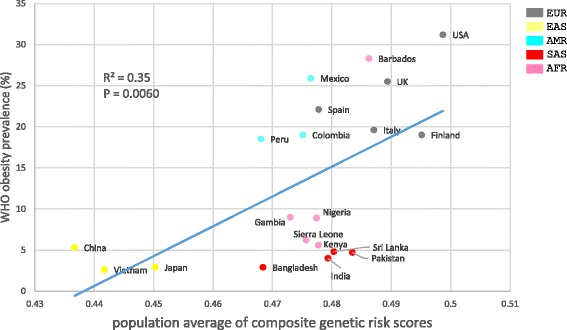
Correlation between WHO-surveyed obesity prevalence and population-level average of composite genetic risk scores

We next explored the correlation between composite genetic risk scores and obesity prevalence surveyed by WHO (Fig. 7). Four European countries and USA have very high obesity rates (≥19%), whereas Vietnam (2.6%) and Japan (2.9%) have the lowest obesity rate. China has an obesity prevalence of 5.3% which is still much lower than the European countries and USA. The high obesity rates in European populations and low rates in East Asian populations coincide with high genetic risk scores in Europeans and low scores in East Asians (Fig. 6), respectively. Over all five continental groups, the population-level average of composite genetic risk scores is significantly positively correlated (R^2^ = 0.35, *P* = 0.0060) with the obesity prevalence (Fig. 7). The significance of the correlation may suggest the validity of our formula (equation 1). However, we recognize that our formula will require further validation.

### Bias analysis

**Fig. 8:**
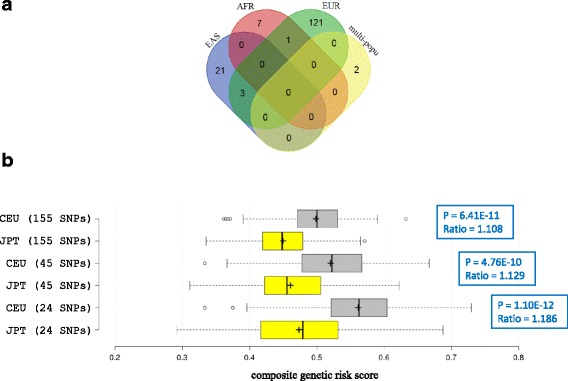
Obesity-associated SNPs detected in different populations and their effects on composite genetic risk scores. (**a**) Overlap of SNPs detected in different populations. In this Venn diagram, each oval represents a population specific set of SNPs, which were detected in GWA studies targeting that population (see Additional file 2: Table S2). For example, the oval for Europe contains 125 SNPs, which indicates that these SNPs were detected in European population-based GWA studies. Multi-population refers to GWA studies performed on mixed ethnic populations (Additional file 2: Table S2). South Asian population-based GWA studies compiled in this report did not result in obesity-associated SNPs reaching genome-wide significance (5 × 10^−8^). (**b**) Comparing distributions of composite genetic risk scores between CEU and JPT populations on different SNP set. Refer to the main text for specifications of three SNP sets (24, 45 and 155 SNPs). In the boxplot, plus symbol indicates average, and center line in the box plot shows the median. Ratio refers to, for each SNP set, the ratio of population-level average between CEU and JPT. P-value is based on the Student’s *t* test

Among the 155 SNPs with GWAS *p*-values less than 5 × 10^−8^ that were used to calculate the composite genetic risk score, 121 (78%) were only detected from GWA studies targeting European populations (Fig. 8a). Naturally, one may raise the concern that the composite score may be biased towards European populations. Indeed, three European populations (CEU, FIN, GBR) ranked top 3 in terms of the average of composite scores (Fig. 6). However, the average score of TSI (Toscani in Italy) is slightly smaller than ITU (Indian Telugu in the UK), while the average score of IBS (Iberian populations in Spain) is below three South Asian populations (STU, PJL, ITU), two African populations (YRI, ACB) and one American population (PUR). Thus, non-European populations could also attain relatively high composite scores. Among the four continental groups except European, East Asian has the most number (24) of obesity-associated SNPs detected from GWA studies (Fig. 8a). If more obesity-associated SNPs being detected from a particular population would make the composite score of that population higher, then the composite scores of East Asian populations would be higher than other three continental group (AFR, SAS, AMR) populations. However, all five East Asian populations ranked lowest in terms of composite score averages and medians (Fig. 6), indicating that the inclusion of more SNPs from a particular population would not necessarily boost that population to a higher genetic risk in comparing with others. It is the allele frequencies, not the number of obesity-associated SNPs, that determine the outcome of composite scores (Additional file 3: Document 1).

Figure 6 clearly shows that East Asian populations have lower obesity risks than Europeans. To further validate this result, we compiled two additional SNP sets. The first set consists of 24 SNPs that were detected from East Asian populations (Fig. 8a). Of these 24 SNPs, three were also detected in European populations. The second set includes these 24 SNPs and additional 21 SNPs that were randomly chosen among the 121 SNPs only detected from European populations (Fig. 8a). Thus, the second set of 45 SNPs would not be obviously biased toward either Europeans or East Asians. We then re-calculated the composite genetic risk scores by using these two SNP sets, respectively, for JPT (Japanese) and CEU populations. Both JPT and CEU ranked first among the East Asian and European populations, respectively (Fig. 6), and both are developed countries. The average of composite scores for CEU is 0.562 for the 24-SNP set, 0.521 for the 45-SNP set and 0.499 for the original 155-SNP set (Fig. 8b). Thus, CEU risk scores actually decreased from the East Asian SNP set to the SNP set dominated by Europeans. On the other hand, the average of composite scores for JPT also slightly decreased. It is 0.474 for the 24-SNP set, 0.461 for the 45-SNP set and 0.450 for the 155-SNP set. Consequently, the ratio of average between CEU and JPT decreased from the East Asian SNP set (1.186) to the original 155-SNP set (1.108). (Figure 8b). The p-vale comparing CEU with JPT averages for the 155-SNP set (6.4 × 10^−11^) became less significant when compared with the *p*-value for the East Asian SNP set (1.1 × 10^−12^) (Fig. 8b). Therefore, the gap between CEU and JPT actually narrows when all 155 SNPs were used. However, the difference is still very significant.

## Discussion

In this study, we explored the worldwide population differentiation in allele frequencies of obesity-associated SNPs. We used hypergeometric model to test whether the effect allele of an obesity-associated SNP was significantly enriched or depleted in each of the 26 populations relative to the global population surveyed in the 1000 Genomes Project [19]. The resulting *p*-values were used to generate an enrichment/depletion heatmap (Fig. 2), which would facilitate the visualization of worldwide allele frequency distributions and help identify patterns. For example, the African populations clearly show a distinct allele enrichment/depletion pattern (Fig. 2). In a conventional approach of using F_st_ (fixation index) to capture the difference in allele frequency between two populations, it would need 325 (26 × 25/2) pairwise comparisons for a single SNP [41]. The F_st_ -based heatmap for more than two hundred obesity SNPs would probably be much more complicated than the one shown in Fig. 2. In addition, an F_st_ score does not correspond to a p-value, and it usually requires the construction of empirical F_st_ distribution (genome-wide or from a random set of SNPs) and then choosing a certain percentile as a significance cutoff. Our hypergeometric approach would need 52 (26 × 2) testings and generate p-values directly. The resulting p-value based heatmap depicts enrichment/depletion patterns of obesity-associated alleles across populations, which may be helpful in providing guidance in implementing population-based interventions. For example, in adults, the allele (A) of the *FTO* variant, rs9939609, increased the risk of obesity in a meta-analysis of pooled populations, but physical activity attenuated this effect [42]. This allele is significantly enriched in the populations with African ancestry but depleted in East Asian populations (Fig. 4b). Thus, the effectiveness of exercise interventions on the obesity management may vary between different populations. In addition, the p-value based heatmap could connect to linkage disequilibrium patterns (Fig. 4).

Among the 225 obesity-associated SNPs collected in this study, 195 (86.7%) possess effect alleles significantly enriched or depleted in at least one of the 26 populations. In extreme cases (Table 2), some SNPs (e.g. rs2890652, rs10150332) have effect alleles that are almost completely wiped out in a continental group, whereas other SNPs (e.g. rs12229654, rs671) have effect alleles that are fixed in multiple continental groups. Thus, it would be important to conduct GWA studies in different ancestry populations. In addition, because there are much fewer GWA studies of obesity in populations of non-European ancestry (Additional file 2: Table S2), it is possible that additional obesity-associated SNPs could be detected in populations such as Africans or East Asians which show the most distinct enrichment/depletion patterns in known obesity alleles (Fig. 3).

In this study, we calculated the composite genetic risk score for obesity at both the individual and population levels. We used all SNPs reaching genome-wide significance to compute the composite scores. Although most of these SNPs were detected from European populations (Fig. 8a), we assumed that these variants would also affect obesity in non-European populations according to [40]. A recent study also supports the generalization of established SNP associations with BMI in diverse ancestral populations [43]. Genome-wide association studies of type 2 diabetes, a metabolic disease closely associated with obesity, in a range of ancestry groups also revealed that most common-variant susceptibility loci are shared across ethnic groups [44, 45]. Additionally, the biological mechanism linking an SNP to complex trait like obesity should, in general, be functioning across populations since we all belong to the same species. We observed that obesity prevalences in American countries (Mexico, Peru, Colombia) are relatively high (Fig. 7), however, there is no SNPs with genome-wide significance originating from GWAS of American populations (Fig. 8a). There is no SNPs with genome-wide significance originating from GWAS of South Asian populations, either. For practical purpose, in order to assess their genetic risk scores, obesity-associated SNPs detected in populations of other continental groups need to be used. It would be reasonable to use all reported genome-wide significant SNPs [40, 43], instead of arbitrarily choosing a subset of theses SNPs, to calculate the composite genetic risk scores for American and South Asian populations and compare their scores with other world populations. Furthermore, through the bias analysis, we demonstrated that the inclusion of more SNPs from a particular population would not necessarily push that population to a higher genetic risk score in comparing with others (Fig. 8). It is the effect allele frequencies of obesity-associated SNPs that determine the outcome of genetic risk scores (Additional file 3: Document 1).

The results of population-level composite scores show that East Asians seem to be genetically less likely to become obese than the other populations (Fig. 6). The obesity prevalence in East Asian countries is indeed very low (Fig. 1). Do these results imply that East Asian people do not need to exercise as frequently as other populations or eat as healthy as possible to control their body weights? The answer is definitely ‘no’. One important reason is that the proportion of Asian people (including Chinese and Japanese) with a high risk of type 2 diabetes and cardiovascular disease is substantial at BMIs lower than the cut-off point of 25 kg/m^2^ that defines overweight in the current WHO classification (obesity ≥30 kg/m^2^) [46]. In other words, the BMI threshold to trigger other diseases for Asian people may be lower than the threshold for other populations.

Complex traits such as obesity result from the combined effects of multiple genetic variants and their interaction with environment. While this study focuses on the genetic risk factors for obesity, it is important to note that environmental factors such as diet, climate, local pathogens and lifestyle also contribute to obesity. The strength of the linear correlation between the population-level average of composite genetic risk scores and obesity prevalence (Fig. 7) indicates that 35% of the variance in the obesity prevalence is predictable from the genetic risk score. Interestingly, Hemani et al*.* reported heritability (*h* ^2^) estimates of 42% for BMI on a sample of 20,240 quasi-independent sibling pairs [47]. In future studies, a more comprehensive formula to predict the obesity risk would incorporate both genetic and environmental factors. Our composite genetic risk score (equation 1) may be used for the genetic part in such a formula.

## Conclusions

Our study shows substantial population differentiation in allele frequencies of obesity-associated SNPs. Our simple formula (equation 1) to calculate the composite genetic risk score can be applied to individuals from different populations by overcoming the effect size weight issue of obesity–associated SNPs, so that genetic risks of different populations can be compared with each other. Our risk score assessment equation for obesity may also be useful in clinical implications. For example, one can assess a person’s obesity risk based on his genotypes over those obesity-associated SNPs. The approach developed in this study should be applicable to other diseases such as hypertension and type 2 diabetes.

## Additional files

Additional file 1: Table S1. Effect allele frequencies in 26 populations for obesity SNPs. The table lists 225 obesity-associated SNPs and their effect allele frequencies in 26 populations surveyed in the 1000 Genomes Project. (XLSX 229 kb)

Additional file 2: Table S2. GWA studies of obesity. The table lists 29 GWA studies of obesity, the major ethnic group in each GWA study, and their references. (DOCX 70 kb)

Additional file 3: Document 1. Population-level average of composite genetic risk scores and allele frequencies. The document illustrates that the population-level average of composite genetic risk scores is identical to the average of effect allele frequencies of obesity-associated SNPs. (DOCX 13 kb)

## Abbreviations

AFR: Africa
*ALDH2*: Aldehyde dehydrogenase 2
AMR: America
*BDNF*: Brain derived neurotrophic factor
BMI: Body-mass index
EAS: East Asia
EUR: Europe
F_st_: Fixation index
*FTO*: Fat mass and obesity associated
FWER: Family-wise error rate
*GALNT10*: polypeptide N-acetylgalactosaminyltransferase 10
GWAS: Genome-wide association study
*KCTD15*: Potassium channel tetramerization domain containing 15
LD: Linkage disequilibrium
NHW: Non-Hispanic white
SAS: South Asia
*SH2B1*: SH2B adaptor protein 1
SNP: Single nucleotide polymorphism
WHO: World Health Organization; Abbreviations of 26 populations (*e.g.* ACB, CEU, CHB, JPT, PUR, YRI) surveyed in the 1000 Genomes Project are listed in Table 1.
