# Supplementary material for: Population differentiation in allele frequencies of obesity-associated SNPs
Source: BMC Genomics. 2017 Nov 10;18:861. doi: 10.1186/s12864-017-4262-9 (PMC5681842; doi:10.1186/s12864-017-4262-9)
Supplement: Supplementary file 2 — GWA studies of obesity. The table lists 29 GWA studies of obesity, the major ethnic group in each GWA study, and their references. (DOCX 70 kb) [file 12864_2017_4262_MOESM2_ESM.docx]

Additional file 2: Table S2. GWA studies of obesity

| First author of the reference and PMID | Reference published year | Major ethnic group in the GWA study |
| --- | --- | --- |
| Berndt SI (PMID: 23563607) [[1](#_ENREF_1)] | 2013 | European |
| Croteau-Chonka DC (PMID: 20966902) [[2](#_ENREF_2)] | 2010 | Filipino |
| Dorajoo R (PMID: 21544081) [[3](#_ENREF_3)] | 2011 | Chinese, Singapore |
| Frayling TM (PMID: 17434869) [[4](#_ENREF_4)] | 2007 | British |
| Graff M (PMID: 23669352) [[5](#_ENREF_5)] | 2013 | European |
| Heard-Costa NL (PMID: 19557197) [[6](#_ENREF_6)] | 2009 | European |
| Heid IM (PMID: 20935629) [[7](#_ENREF_7)] | 2010 | European |
| Hinney A (PMID: 18159244) [[8](#_ENREF_8)] | 2007 | German |
| Jiao H (PMID: 21708048) [[9](#_ENREF_9)] | 2011 | Swedish |
| Liu CT (PMID: 23966867) [[10](#_ENREF_10)] | 2013 | African |
| Liu JZ (PMID: 20397748) [[11](#_ENREF_11)] | 2010 | Australian |
| Loos RJ (PMID: 18454148) [[12](#_ENREF_12)] | 2008 | European |
| Meyre D (PMID: 19151714) [[13](#_ENREF_13)] | 2009 | European |
| Monda KL (PMID: 23583978) [[14](#_ENREF_14)] | 2013 | African |
| Namjou B (PMID: 24348519) [[15](#_ENREF_15)] | 2013 | European |
| Ng MC (PMID: 21701570) [[16](#_ENREF_16)] | 2011 | African |
| Okada Y (PMID: 22344221) [[17](#_ENREF_17)] | 2012 | East Asian |
| Pei YF (PMID: 24064335) [[18](#_ENREF_18)] | 2013 | Multiple populations |
| Scannell Bryan M (PMID: 25133637) [[19](#_ENREF_19)] | 2014 | Bangladeshi |
| Scherag A (PMID: 20421936) [[20](#_ENREF_20)] | 2010 | European |
| Speliotes EK (PMID: 20935630) [[21](#_ENREF_21)] | 2010 | European |
| Thorleifsson G (PMID: 19079260) [[22](#_ENREF_22)] | 2008 | European |
| Wang K (PMID: 21552555) [[23](#_ENREF_23)] | 2011 | European |
| Wang KS (PMID: 22446040) [[24](#_ENREF_24)] | 2012 | European |
| Wen W (PMID: 22344219) [[25](#_ENREF_25)] | 2012 | East Asian |
| Wen W (PMID: 24861553) [[26](#_ENREF_26)] | 2014 | East Asian |
| Wheeler E (PMID: 23563609) [[27](#_ENREF_27)] | 2013 | European |
| Willer CJ (PMID: 19079261) [[28](#_ENREF_28)] | 2008 | European |
| Yang J (PMID: 22982992) [[29](#_ENREF_29)] | 2012 | European |

**References**

1. Berndt, S.I., et al., *Genome-wide meta-analysis identifies 11 new loci for anthropometric traits and provides insights into genetic architecture.* Nat Genet, 2013. **45**(5): p. 501-12.

2. Croteau-Chonka, D.C., et al., *Genome-wide association study of anthropometric traits and evidence of interactions with age and study year in Filipino women.* Obesity, 2011. **19**(5): p. 1019-27.

3. Dorajoo, R., et al., *Replication of 13 obesity loci among Singaporean Chinese, Malay and Asian-Indian populations.* Int J Obes, 2012. **36**(1): p. 159-63.

4. Frayling, T.M., et al., *A common variant in the FTO gene is associated with body mass index and predisposes to childhood and adult obesity.* Science, 2007. **316**(5826): p. 889-94.

5. Graff, M., et al., *Genome-wide analysis of BMI in adolescents and young adults reveals additional insight into the effects of genetic loci over the life course.* Hum Mol Genet, 2013. **22**(17): p. 3597-607.

6. Heard-Costa, N.L., et al., *NRXN3 is a novel locus for waist circumference: a genome-wide association study from the CHARGE Consortium.* PLoS Genet, 2009. **5**(6): p. 26.

7. Heid, I.M., et al., *Meta-analysis identifies 13 new loci associated with waist-hip ratio and reveals sexual dimorphism in the genetic basis of fat distribution.* Nat Genet, 2010. **42**(11): p. 949-60.

8. Hinney, A., et al., *Genome wide association (GWA) study for early onset extreme obesity supports the role of fat mass and obesity associated gene (FTO) variants.* PLoS One, 2007. **2**(12).

9. Jiao, H., et al., *Genome wide association study identifies KCNMA1 contributing to human obesity.* BMC Med Genomics, 2011. **4**(51): p. 1755-8794.

10. Liu, C.T., et al., *Genome-wide association of body fat distribution in African ancestry populations suggests new loci.* PLoS Genet, 2013. **9**(8): p. 15.

11. Liu, J.Z., et al., *Genome-wide association study of height and body mass index in Australian twin families.* Twin Res Hum Genet, 2010. **13**(2): p. 179-93.

12. Loos, R.J., et al., *Common variants near MC4R are associated with fat mass, weight and risk of obesity.* Nat Genet, 2008. **40**(6): p. 768-75.

13. Meyre, D., et al., *Genome-wide association study for early-onset and morbid adult obesity identifies three new risk loci in European populations.* Nat Genet, 2009. **41**(2): p. 157-9.

14. Monda, K.L., et al., *A meta-analysis identifies new loci associated with body mass index in individuals of African ancestry.* Nat Genet, 2013. **45**(6): p. 690-6.

15. Namjou, B., et al., *EMR-linked GWAS study: investigation of variation landscape of loci for body mass index in children.* Front Genet, 2013. **4**(268).

16. Ng, M.C., et al., *Genome-wide association of BMI in African Americans.* Obesity, 2012. **20**(3): p. 622-7.

17. Okada, Y., et al., *Common variants at CDKAL1 and KLF9 are associated with body mass index in east Asian populations.* Nat Genet, 2012. **44**(3): p. 302-6.

18. Pei, Y.F., et al., *Meta-analysis of genome-wide association data identifies novel susceptibility loci for obesity.* Hum Mol Genet, 2014. **23**(3): p. 820-30.

19. Scannell Bryan, M., et al., *Genome-wide association studies and heritability estimates of body mass index related phenotypes in Bangladeshi adults.* PLoS One, 2014. **9**(8).

20. Scherag, A., et al., *Two new Loci for body-weight regulation identified in a joint analysis of genome-wide association studies for early-onset extreme obesity in French and german study groups.* PLoS Genet, 2010. **6**(4): p. 1000916.

21. Speliotes, E.K., et al., *Association analyses of 249,796 individuals reveal 18 new loci associated with body mass index.* Nat Genet, 2010. **42**(11): p. 937-48.

22. Thorleifsson, G., et al., *Genome-wide association yields new sequence variants at seven loci that associate with measures of obesity.* Nat Genet, 2009. **41**(1): p. 18-24.

23. Wang, K., et al., *A genome-wide association study on obesity and obesity-related traits.* PLoS One, 2011. **6**(4): p. 0018939.

24. Wang, K.S., et al., *A novel locus for body mass index on 5p15.2: a meta-analysis of two genome-wide association studies.* Gene, 2012. **500**(1): p. 80-4.

25. Wen, W., et al., *Meta-analysis identifies common variants associated with body mass index in east Asians.* Nat Genet, 2012. **44**(3): p. 307-11.

26. Wen, W., et al., *Meta-analysis of genome-wide association studies in East Asian-ancestry populations identifies four new loci for body mass index.* Hum Mol Genet, 2014. **23**(20): p. 5492-504.

27. Wheeler, E., et al., *Genome-wide SNP and CNV analysis identifies common and low-frequency variants associated with severe early-onset obesity.* Nat Genet, 2013. **45**(5): p. 513-7.

28. Willer, C.J., et al., *Six new loci associated with body mass index highlight a neuronal influence on body weight regulation.* Nat Genet, 2009. **41**(1): p. 25-34.

29. Yang, J., et al., *FTO genotype is associated with phenotypic variability of body mass index.* Nature, 2012. **490**(7419): p. 267-72.
