# Supplementary material for: Population differentiation in allele frequencies of obesity-associated SNPs
Source: BMC Genomics. 2017 Nov 10;18:861. doi: 10.1186/s12864-017-4262-9 (PMC5681842; doi:10.1186/s12864-017-4262-9)
Supplement: Supplementary file 3 — Population-level average of composite genetic risk scores and allele frequencies. The document illustrates that the population-level average of composite genetic risk scores is identical to the average of effect allele frequencies of obesity-associated SNPs. (DOCX 13 kb) [file 12864_2017_4262_MOESM3_ESM.docx]

**Supplementary Document 1**

**Population-level average of composite genetic risk scores and allele frequencies**

Let’s use a simple example in which there are 3 obesity-associated SNPs and the population size is 4.

First, according to the equation 1 in the main text, an individual’s composite genetic risk score is, (X_1_ + X_2_ + X_3_) / (2*3), where X_i_ refers to copies of risk alleles at the i^th^ SNP.

Then, the population-level average of composite genetic risk scores can be calculated as

Popu_avg_score = 1/4 * [(X_1,1_ + X_2,1_ + X_3,1_) / (2*3) + (X_1,2_ + X_2,2_ + X_3,2_) / (2*3) + (X_1,3_ + X_2,3_ + X_3,3_) / (2*3) + (X_1,4_ + X_2,4_ + X_3,4_) / (2*3) ]

= 1/3 * [(X_1,1_ + X_1,2_ + X_1,3_ + X_1,4_) / (2*4) + (X_2,1_ + X_2,2_ + X_2,3_ + X_2,4_) / (2*4) + (X_3,1_ + X_3,2_ + X_3,3_ + X_3,4_) / (2*4) ]

= 1/3 * [AF_1_ + AF_2_ + AF_3_]

In the above formula, X_i,j_ refers to copies of risk alleles (X_i,j_ $\in$ {0,1,2}) at the i^th^ SNP of j^th^ individual; AF_i_ is the risk allele frequency of i^th^ SNP in this population. Thus, the population-level average of composite genetic risk scores is equal to the average of risk allele frequencies over the three obesity-associated SNPs.

In general, for *m* obesity-associated SNPs and the population size of *n*, we can also use the above procedure to illustrate that

Popu_avg_score = 1/m * [AF_1_ + AF_2_ + … + AF_m_]

which is the average of risk allele frequencies over *m* obesity-associated SNPs.
